# Rheological and Interaction Analysis of Asphalt Binder, Mastic and Mortar

**DOI:** 10.3390/ma12010128

**Published:** 2019-01-02

**Authors:** Meng Chen, Barugahare Javilla, Wei Hong, Changluan Pan, Martin Riara, Liantong Mo, Meng Guo

**Affiliations:** 1 State Key Laboratory of Silicate Materials for Architectures, Wuhan University of Technology, Wuhan 430070, China; 265797@whut.edu.cn (M.C.); makorogo@whut.edu.cn (B.J.); hw0238@whut.edu.cn (W.H.); panchangluan@whut.edu.cn (C.P.); m.m.riara@whut.edu.cn (M.R.); 2 Department of Physical sciences, South Eastern Kenya University, Kitui 170-90200, Kenya; 3 College of Architecture and Civil Engineering, Beijing University of Technology, Beijing 100124, China; gm@bjut.edu.cn

**Keywords:** bitumen, mastic, mortar, rheological properties, physical interaction

## Abstract

This paper investigated the rheological properties of asphalt binder, asphalt mastic and asphalt mortar and the interaction between asphalt binder, mineral filler and fine aggregates. Asphalt binder, mastic and mortar can be regarded as the binding phase at different scales in asphalt concrete. Asphalt mastic is a blend of asphalt binder and mineral filler smaller than 0.075 mm while asphalt mortar consists of asphalt binder, mineral filler and fine aggregate smaller than 2.36 mm. The material compositions of mastic and mortar were determined from the commonly used asphalt mixtures. Dynamic shear rheometer was used to conduct rheological analysis on asphalt binder, mastic and mortar. The obtained test data on complex modulus and phase angle were used for the construction of rheological master curves and the investigation of asphalt-filler/aggregate interaction. Test results indicated a modulus increase of three- to five-fold with the addition of filler and a further increase of one to two orders of magnitude with cumulative addition of fine aggregates into asphalt binder. Fine aggregates resulted in a phase change for mortar at high temperatures and low frequencies. The filler had stronger physical interaction than fine aggregate with an interaction parameter of 1.8–2.8 and 1.15–1.35 respectively. Specific area could enhance asphalt-filler interaction. The mastic and mortar modulus can be well predicted based on asphalt binder modulus by using particle filling effect. Asphalt mortar had a significant modulus reinforcement and phase change and thus could be the closest subscale in terms of performance to that of asphalt mixtures. It could be a vital scale that bridges the gap between asphalt binder and asphalt mixtures in multiscale performance analysis.

## 1. Introduction

By the end of 2017, the total length of highways in China open to traffic reached 477 million km with a highway density of 19.5 km per 100 km^2^. Among these highways, 136.5 thousand kilometers of expressways have been built according to advanced modern transportation standard. Up to 90% of high-grade highways in China are asphalt pavements. These asphalt pavements usually consist of a three-layer structure comprising the wearing course layer, intermediate binder course layer and base course layer from top to down respectively. Most of the asphalt pavements are designed to have a service life of about 15 years for high-grade highways. However, large amount of maintenance and repair works are needed before reaching the design life. Permanent deformation or rutting, moisture damage, reflective cracking, low temperature cracking and fatigue cracking are the main distresses addressed during maintenance [1,2,3,4]. Currently, it has become a big challenge to design more durable asphalt pavements to deal with the increasing traffic volume of modern transportation and extreme climate change in the future. 

Asphalt pavements are usually paved with hot mix asphalt which consists of coarse aggregates, fine aggregates, filler and bitumen as the binder. Each of these components has a great influence on the performance of asphalt mixtures after they have been paved and compacted [5,6,7]. In general, coarse aggregates form the stone skeleton while fine aggregate, mineral filler and asphalt binder fill in the voids within the coarse aggregates and also hold the stone skeleton as a whole. Depending on the difference of scale, the binding phase is usually divided in to three levels: pure asphalt binder, asphaltic mastic (a blend of asphalt binder and mineral filler smaller than 0.075 mm) and asphaltic mortar (a matrix that consists of fine aggregate smaller than 2.36 mm, mineral filler and asphalt binder). The stone skeleton of coarse aggregates provides the main contribution of loading bearing and rutting resistance, while the binding phases strongly relate to the problems of aging, fatigue, cracking, and moisture damage [8,9].

In order to design better and more durable asphalt mixtures, the fundamental properties of asphalt mixtures should be predictable by an upscaling procedure based on the properties of asphalt binder, mastic and mortar [7,10]. This requires that a correlation exists between the test results of asphalt binder, mastic and mortar scales and those of asphalt mixtures. Because of the exclusion of mineral fractions, a gap exists between the properties of pure asphalt binder and those of asphalt mixtures. Asphalt mastic is usually considered as the actual binding component in the mix which coats the coarse aggregates [11]. Apart from coarse aggregates, asphalt mastic is further blended with fine fractions of aggregate to form the mortar that fills in the voids within the coarse aggregates. Therefore, asphalt mortar is the sub scale close to asphalt mixture [12]. In general, asphaltic mortar can bridge the gap of scale between bitumen and asphalt mixture.

Asphalt binder can be reinforced by the addition of mineral filler. Further reinforcement is obtained by the addition of fine aggregates [13,14,15]. In general, the mastic and mortar system can be regarded as two-phase composite materials, in which, asphalt binder is the matrix phase and mineral particles are the reinforcement phase. The interaction between asphalt binder, filler and fine fractions was found to be a physical process and could be explained by the mechanisms of particle reinforcement [14,15,16]. Fillers and fine aggregate had significant influences on rheological properties of the corresponding mastic and mortar. Finally, they affect the performances of asphalt mixture at both high and low temperatures. 

In recent years, many researches have been done on asphalt mastic with various types of filler including Portland cement [17,18,19], hydrated lime [17,19,20,21], fly ash [20,22], natural and synthetic zeolites derived from fly ash [23,24,25,26], volcanic ash [27], oil shale ash [28], rice straw ash [29], red mud [30,31], limestone dust [19], glass powder [31], brick dust [31], carbide lime [31], copper tailings [21,31], natural bentonite clay [32], ladle furnace slag [33], silica fume [21], magnetite [34], waste stone sawdust [35] and steel slag [36]. Asphalt mixtures prepared using the same content of bitumen but different waste materials as fillers (glass powder, limestone dust, red mud, rice straw ash, brick dust, carbide lime and copper tailings) showed satisfactory mechanical and volumetric properties. In particular, fine fillers such as limestone dust and red mud had a significant positive effect on the stiffness and cracking resistance of the asphalt mixtures. Free energy of adhesion between bitumen and aggregates was improved by stone powder fillers but use of hydrated lime, calcium carbonate and Portland cement had a negative effect on the adhesion energy. Recycled ladle furnace slag is beneficial to the volumetric properties, stiffness, indirect tensile strength and resistance to dynamic loading of asphalt concrete. Active filler including hydrated lime and cement had the potential to improve the resistance to moisture damage [17,18,19]. The usage of micro filler and nano-clay had shown high reinforcing potential in bitumen mastics [27,32]. Many waste materials were used as substitutes to natural limestone filler due to the consideration of recycling and environmental protection. Magnetite filler could be exploited for induction or microwave healing of asphalt pavement cracks [34]. Regardless of the type of filler to be used, high interaction or compatibility between asphalt binder and filler is always desired [37,38,39,40,41,42,43]. Diab reported that there was no proof of chemical mechanisms between bitumen and various fillers including blast-furnace slag, silica fume, fly ash, and hydrated lime. Nonlinear rheological properties could differentiate the performance of different types of mastic [22]. Guo’s research indicated that the interfacial interaction between asphalt binder and filler strongly depended on the diffusion behavior of asphalt binder components as well as the chemical composition of mineral fillers [15,43,44]. The reinforcement effect of filler was widely investigated by means of rheological properties including complex modulus, phase angle, creep recovery and stiffness. Various indices were proposed to evaluate the asphalt-filler interaction ability. Liu reported that the evaluation index based on phase angle was more sensitive than that based on complex modulus. Temperature and specific surface area were the two main factors effecting the interfacial interaction between asphalt binders and mineral fillers [14,45,46,47].

With respect to asphaltic mortar, recycled waste materials such as gneiss, ceramsite, ceramic, marble, redbrick ash, steel slag, fine dune sand, and river sand were reported as a possible substitute for fine aggregates [48,49]. It was found that the morphological characteristics of fine aggregates (i.e., shape factors, angularity, and surface texture) significantly affect the mechanical performance of asphalt mixtures [50]. The increase of fine aggregate content was found to jeopardize the resistance of asphalt mixture on low-temperature cracking [51]. Fine aggregates had a more significant impact on skid resistance on the macro-texture level than micro-texture level [52,53]. Li reported that the complex modulus values between asphaltic mastic and mortar were highly correlated and the dissipated energy method could well explain the fatigue performance of asphalt materials at different scales [6]. 

In the recent years, various types of filler and fine aggregates have been used as a substitute for traditional mineral components in asphalt mixtures. The associated asphalt mixtures were reported to have a comparable performance with control ones based on laboratory testing. However, the long-term pavement performance of these non-traditional asphalt mixtures is needed for validation. Rutting and moisture damage was frequently found among the premature distresses of asphalt pavements in China. Improper use of filler and fine aggregate worsened the resistance of rutting and moisture damage. This paper aimed to get insight into the correlation of the rheological property and the degree of physical interaction between asphalt binder, mastic and mortar over a wide range of temperatures (30 °C to 70 °C) and frequencies (0.1 rad/s to 400 rad/s). The sensitivity of common parameters used to analyze the interaction among asphalt binder, filler and fine aggregate was also assessed. In addition, the asphalt materials subscale whose interaction was more representative for a multiscale performance research was established. The material components of the studied asphaltic mastic and mortar were determined from the commonly used asphalt mixtures in asphalt pavements. This ensured that the used asphaltic mastic and mortar were consistent with those applied in actual asphalt pavements. The rheological properties of asphalt binder, mastic and mortar were investigated by means of dynamic shear rheometer. The interaction between asphalt binder, filler and fine aggregate was evaluated based on the change of complex modulus and phase angle. Several interaction parameters proposed by particle reinforcement theory were applied for interaction evaluation and validation. 

## 2. Materials and Methods

### 2.1. Materials

Neat bitumen (90#) and SBS (Styrene-Butadiene-Styrene) polymer modified bitumen were used. Both asphalt binders were supplied by Panjin Northern Asphalt Co. Ltd, Panjin, China and the content of SBS polymer in the modified asphalt binder was 4.5%. SBS modified bitumen is commonly used in the construction of the surface wearing course and intermediate layer, while neat bitumen 90# is used in the bottom layer of most three-layer asphalt pavements in northern China. Table 1 shows the basic properties of the asphalt binders.

Limestone and basalt aggregates were selected for this study. AC-13 asphalt mixtures designed for the surface wearing course layer contained basalt aggregates while AC-20 asphalt mixture designed for the intermediate binder course layer and AC-25 asphalt mixture for the base course layer contained limestone aggregates. 13, 20 and 25 are the nominal maximum aggregate sizes. More detailed information on the related properties of the used aggregate and filler can be found in our previous paper [54]. The gradation limits of these three mixtures were designed according to JTG F40-2004 [55] and the combined aggregates grading can be found in Table 2. The optimum bitumen contents of AC-13, AC-20 and AC-25 asphalt mixtures were 4.7%, 4.3% and 3.9% respectively as determined by Marshall method with 75 blows per face. These bitumen contents were in agreement with the practical application values in the field.

Based on the mixture compositions listed in Table 2, the corresponding compositions for mastic and mortar fractions were determined as shown in Table 3 and Table 4. Asphalt mastic was determined by the combination of bitumen content and filler content. Asphalt mortar was a mixture of fine aggregates, filler and asphalt binder. Depending on the maximum size of the fine aggregates, three different levels of the mortar scale were investigated. The highest, middle and lowest mortar scales contained fine aggregates with a maximum size of 2.36 mm, 1.18 mm and 0.6 mm respectively. Asphalt mastic and mortar specimens were made following an optimized protocol to obtain homogeneous mixtures. The compositions of these asphaltic materials were heated in the oven at 170 °C for 2 h, after which they were blended until they became homogeneous.

### 2.2. Test Methods 

DSR (MCR101, Anton Paar, Graz, Germany) was used to test the rheological properties of asphalt binder, mastic and mortar specimens. DSR test samples were sandwiched between two circular plates of diameter 25 mm. The lower plate was fixed while the upper plate oscillated back and forth, at frequencies ranging from 0.01 rad/s to 400 rad/s and at temperatures between 30 °C and 70 °C. Asphalt mastic was tested based on the same protocols as bitumen. For mortar samples, due to the addition of fine aggregates, the gap height between the parallel plates was designed to be a minimum of three times the size of the largest fine aggregates in the sample. For that case, asphalt mortar samples containing 0.6, 1.18 and 2.36 mm fine aggregates needed a gap height of 2 mm, 4 mm and 7 mm respectively as illustrated in Table 5. 

Circular mortar test specimens were made by pouring hot and well blending mortar into silicon rubber mounds with a diameter 25 mm and depth of 2, 4 and 7 mm depending on the maximum size of fine aggregates. In order to improve the interface adhesion between the mortar specimen and DSR plates, super glue was used for bonding specimens to the DSR plates. To do so, the zero gap calibration of the DSR plates was first carried out. Super glue was spread on the surfaces of the bottom and upper plates. The mortar specimens were removed from the silicon rubber mold and carefully placed on the bottom plate. The upper plate was lowered and the mortar specimens were squeezed to the desired thickness. DSR frequency sweep testing on asphalt binder, mastic and mortar samples was carried out on five test temperatures including 30 °C, 40 °C, 50 °C, 60 °C, and 70 °C and frequencies from 0.1 rad/s to 400 rad/s. The obtained complex modulus and phase angle was used to investigate the rheological properties of various asphaltic materials and the interaction between asphalt binder, filler and fine aggregates.

## 3. Results and Discussion

### 3.1. Master Curves of Various Asphalt Materials

**Figure 1:**
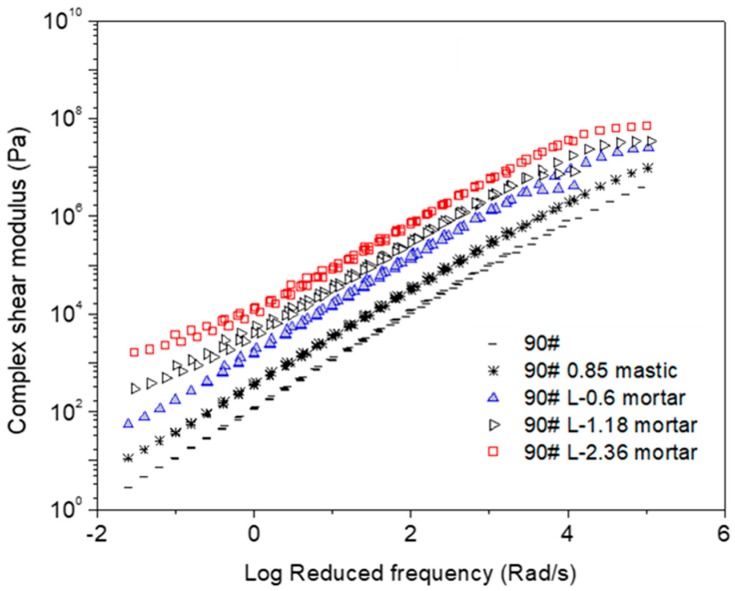
Complex modulus master curves of AC-25 based asphaltic materials including neat asphalt binder, mastic and mortar at a reference temperature of 60 °C.

**Figure 2:**
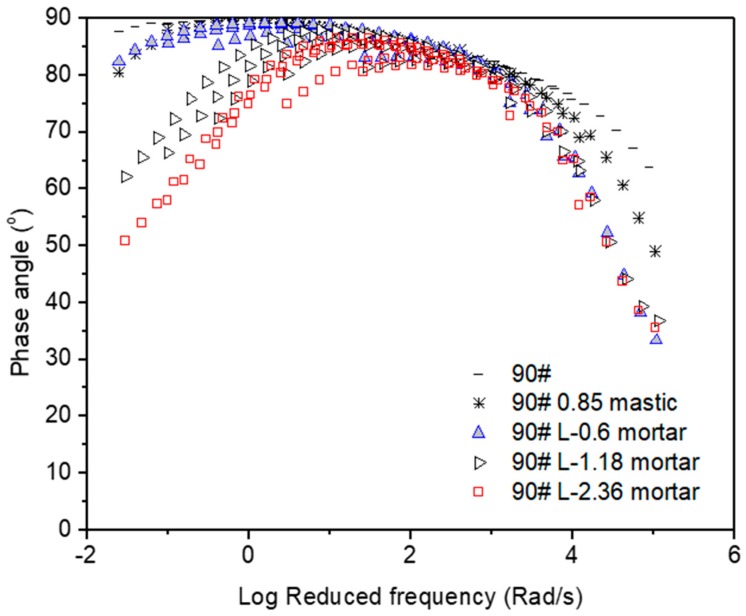
Phase angle master curves of AC-25 based asphaltic materials including neat asphalt binder, mastic and mortar at a reference temperature of 60 °C.

**Figure 3:**
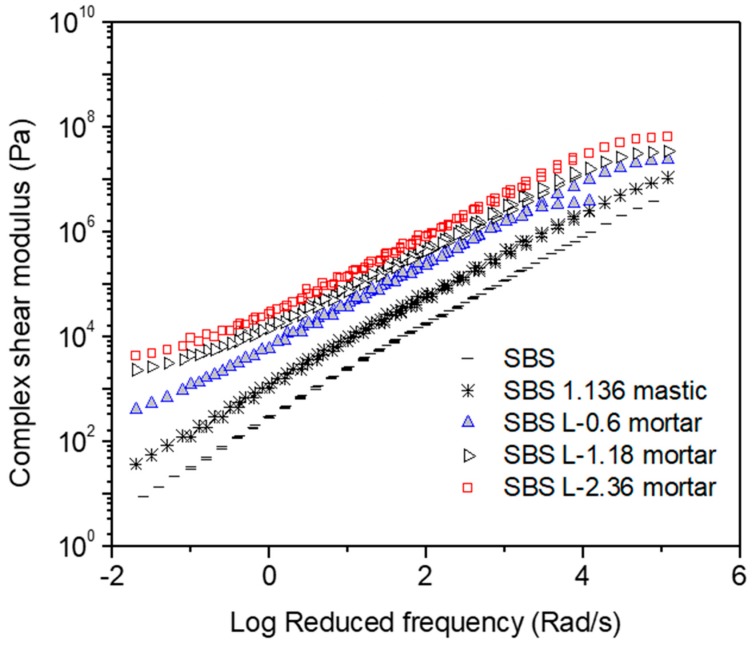
Complex modulus master curves of AC-20 based asphaltic materials including SBS modified asphalt binder, mastic and mortar at a reference temperature of 60 °C.

**Figure 4:**
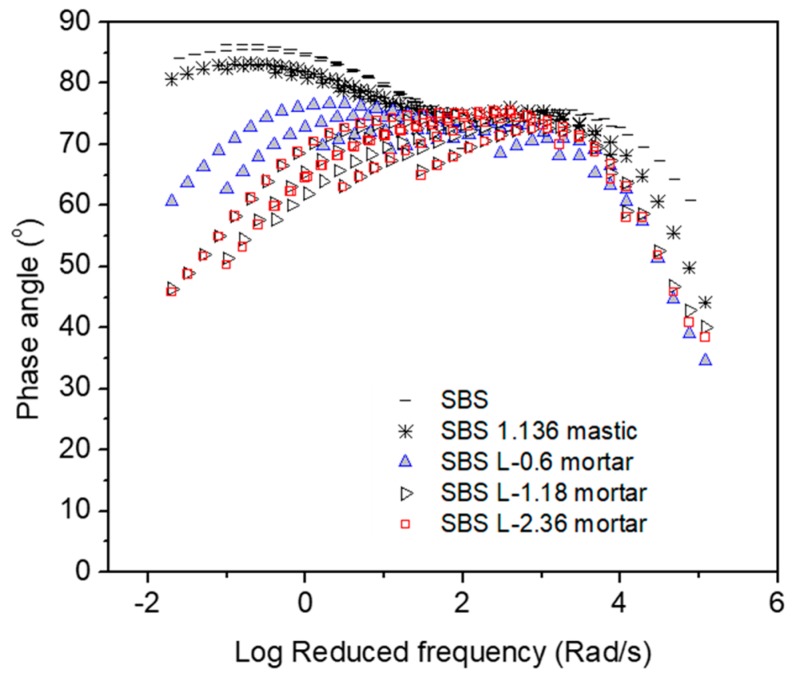
Phase angle master curves of AC-20 based asphaltic materials including neat asphalt binder, mastic and mortar at a reference temperature of 60 °C.

**Figure 5:**
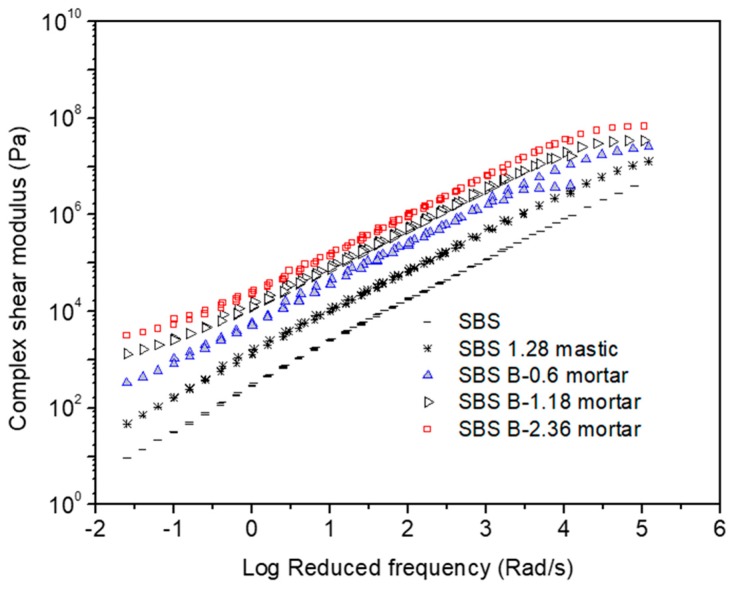
Complex modulus master curves of AC-13 based asphaltic materials including SBS modified asphalt binder, mastic and mortar at a reference temperature of 60 °C.

**Figure 6:**
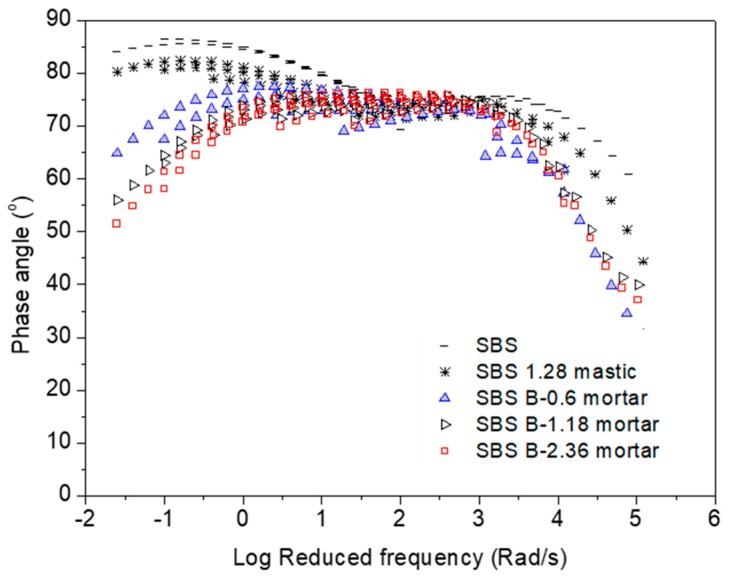
Phase angle master curves of AC-13 based asphalt materials including SBS modified asphalt binder, mastic and mortar at a reference temperature of 60 °C.

Figure 1, Figure 2, Figure 3, Figure 4, Figure 5 and Figure 6 present the master curves of various asphalt materials at a reference temperature of 60 °C. The master curves were constructed by means of the time-temperature superposition principle (TTSP). The complex modulus and phase angle data obtained at different temperatures including 30 °C, 40 °C, 50 °C, 60 °C and 70 °C, were horizontally shifted to the reference temperature of 60 °C to form a smooth master curve. By doing this, the rheological properties indicated by complex modulus and phase angle were explained over a wide range of reduced frequencies. Data at low frequencies represent the properties at high temperature (e.g., 70 °C). On the contrary, those at high frequencies indicate the properties at low temperature (e.g., 30 °C). 

Figure 1 shows various master curves of complex modulus obtained from AC-25 based asphaltic materials including neat asphalt binder, mastic and mortar. The differences are distinct between neat asphalt binder, mastic and mortars containing different sizes of fine aggregates. Compared with the neat asphalt binder, the addition of filler made an obvious modulus increase of the resultant mastic. Further addition of fine aggregate led to a higher modulus for mortar. Increasing the maximum size of fine aggregates also resulted in an modulus increase of mortar. In general, mastic had a threefold increase in complex modulus compared with neat asphalt binder. However, depending on the size of fine aggregate, mortar could have an increase of one to two orders of magnitude on complex modulus. The huge increase in complex modulus of mortar helps to improve the rutting resistance of asphalt mixtures at high temperature.

Figure 2 presents the master curves of phase angle obtained from AC-25 based asphaltic materials including neat asphalt binder, mastic and mortar. Among these asphalt materials, a significant change can be seen at low reduced frequencies, which is equal to high temperature of 70 °C. The phase angle tended to drop down with the addition of filler and fine aggregates. Larger size of fine aggregates led to a more obvious reduction on phase angle at the range of high frequency. It should be noted that phase angle is an index for the ratio between viscosity and elasticity. A purely viscous liquid and an ideal elastic solid have a phase angle of 90° and 0°, respectively. Neat asphalt binder behaved like a purely viscous liquid with a phase angle close to 90° at very high temperatures. Asphalt mastic only deviated a little from neat asphalt binder. However, the addition of fine aggregate could result in a significant change on phase angle, which was strongly indicated by a phase change from a viscous to viscoelastic range. In general, low phase angle and viscoelastic behavior is desired for improving rutting resistance of asphalt mixtures. Combining the data obtained on complex modulus and phase angle, it can be seen that the corresponding material components in asphalt mixture including asphalt binder, mastic and mortar behaved differently at high temperatures. The addition of filler and fine aggregates could result in higher complex moduli and lower phase angles as well as phase change from a viscous to viscoelastic range.

Figure 3 presents the master curves of complex modulus obtained from AC-20 based asphalt materials. AC-20 asphalt mixture is commonly used as the intermediate layer in typical three-layer asphalt pavements in China. The intermediate layer is considered to be subjected to disadvantageous shear stress under wheal loadings and thus susceptible to rutting at high temperatures. Field inspection demonstrated that the intermediate layer contributed most of permanent deformation of asphalt pavements with a semi-rigid base. In order to increase the rutting resistance, SBS modified asphalt binder is commonly used instead of neat asphalt binder together with a stronger coarse stone skeleton containing fewer fine fractions for the intermediate layer. In Figure 3, the material composition of the studied mastic and mortar was determined from the aggregate gradation and asphalt binder content of a typical AC-20 mixture. Similarly, SBS modified asphalt mastic and mortars all have higher complex modulus compared with SBS modified asphalt binder. In general, the addition of filler into SBS modified asphalt binder quadrupled the complex modulus of mastic. Further addition of fine aggregates resulted in a more significant improvement on complex modulus of mortar. Increasing the size of fine aggregate from 0.6 mm to 2.36 mm led to three-fold increase in complex modulus.

Figure 4 gives a summary of the master curves of phase angle obtained from AC-20 based asphalt materials. SBS modified asphalt binder, mastic and mortars containing different fine aggregate maximum sizes were included. The change tendency of asphalt mastic is close to that of SBS modified asphalt binder with a slight reduction over the wide range of reduced frequencies. The addition of fine aggregates resulted in an obvious shift of phase angle, thus confer more elastic property on asphalt mortar. This helped to transfer the visco characteristic of bitumen to a viscoelastic characteristic of mortar at high temperatures. Therefore, the actual fine fractions improve the rutting resistance of the AC-20 mixture. 

Figure 5 and Figure 6 give the master curves of complex modulus and phase angle of AC-13 based asphalt materials including neat asphalt binder, mastic and mortar. AC-13 asphalt mixture is widely used in the construction of surface wearing course in China. Basalt aggregates are commonly used instead of limestone aggregates in order to improve surface abrasion resistance. It is observed that the change tendencies of AC-13 based asphalt materials were similar to those of AC-20 and AC-25 based asphalt mixtures, which were presented in Figure 1 and Figure 3 respectively. The hardening effect on complex modulus can be well distinguished by the addition of filler and fine basalt aggregates. With respect to phase angle, the addition of basalt fine aggregates can result in a similar phase change at low reduced frequencies, which is an equivalent to high temperature.

**Figure 7:**
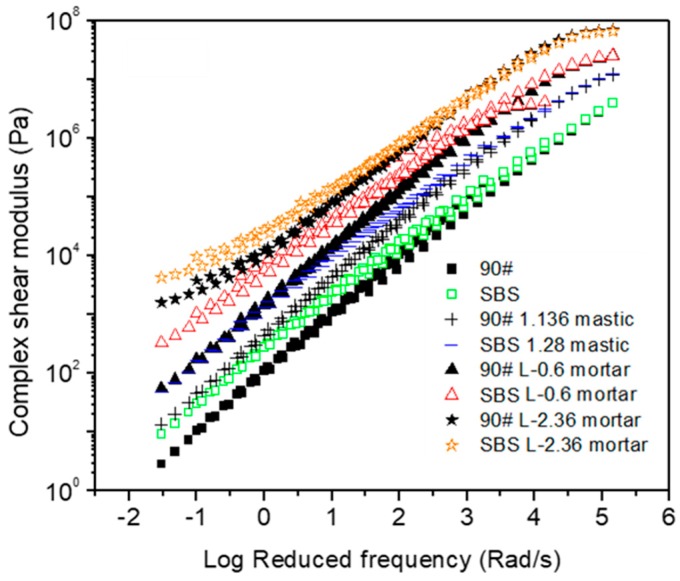
Complex modulus comparison between 90# neat asphalt and SBS modified asphalt based materials at a reference temperature of 60 °C.

**Figure 8:**
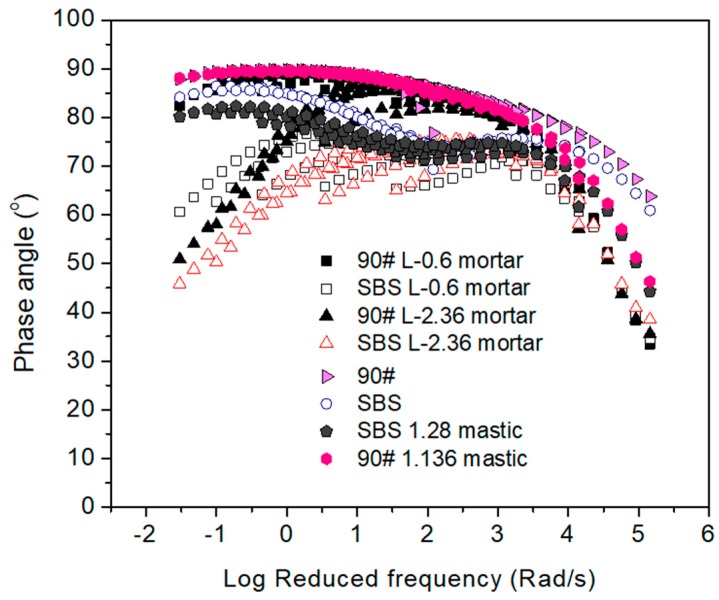
Phase angle comparison between 90# neat asphalt and SBS modified asphalt based materials at a reference temperature of 60 °C.

Figure 7 and Figure 8 show the influence of type of asphalt binder on complex modulus and phase angle, respectively. Compared with neat asphalt binder, SBS modified asphalt materials had an advantage on complex modulus at low reduced frequencies regardless of the scale of material. It also indicated that mortar made by neat asphalt binder combined with larger fine aggregates (for example, 2.36 mm) could be comparable to mortar that consists of SBS modified asphalt binder and smaller fine aggregate (for example, 0.6 mm). This indicated that well-designed material composition of asphalt mortar could be important for performance improvement. With respect to phase angle, a plateau was observed for SBS modified asphalt binder, but not for neat asphalt binder materials. This strongly indicated the existence of the polymer network. In general, SBS modification could result in a small reduction of phase angle. This effect was relatively limited when compared with neat asphalt binder at low reduced frequencies. The effect of filler is not obvious for either neat asphalt binder or SBS modified asphalt binder. However, fine aggregates could be decisive for asphalt mortar. The interaction between fine aggregates tended to result in a phase change, which is considered to benefit the rutting resistance of asphalt mixtures at high temperatures. Based on the analysis above, it could be concluded that the use of polymer modified asphalt binder and coarser fine aggregate should be of interest to improve the rheological properties of mortar at high-temperatures. 

### 3.2. Interaction Analysis between Asphalt Binder, Filler and Fine Aggregate

Asphalt mastic and mortar can be regarded as two-phase particle reinforced composite materials. In this system, asphalt binder is the matrix phase, while filler and fine aggregates acts as the reinforcement phase. Both the filler and fine aggregates can be defined as rigid particles when considering the huge modulus difference between asphalt binder and mineral stone. The addition of filler and fine aggregates into asphalt binder results in modulus reinforcement. The interaction between asphalt binder and mineral particles could be expressed in terms of phase angle using the following equation:

(1) $${\tan\delta }_{c}=\left(1-\emptyset_{f}\right)\left(1+A\right){\tan\delta }_{m},$$

where: $\delta_{c}$ and $\delta_{m}$ are the phase angles of asphalt based composite material and the asphalt matrix, respectively; $\emptyset_{f}$ is the volume fraction of the reinforcement phase. A is the interaction parameter between the asphalt matrix phase and reinforcement phase.

Since the phase angle of mastic/mortar and asphalt binder can be tested, the interaction parameter between asphalt binder and mineral particles can be calculated by using:

(2) $$A=\frac{{\tan\delta }_{c}}{{\tan\delta }_{m}\left(1-\emptyset_{f}\right)}-1,$$

It should be noted that a lower value of the A interaction parameter indicates a stronger interaction between asphalt binder and mineral particles. 

K.D. Ziegel et al. proposed Equation (3) to estimate the loss tangent of composite materials as the function of filler volume fraction [16]:

(3) $${\tan\delta }_{c}=\frac{{\tan\delta }_{m}}{1+1.5\emptyset_{f}B},$$

where: B is the interaction parameter that represents the matrix phase and reinforcement phase interaction ability. A higher value of B indicates a stronger interaction between the matrix phase and reinforcement phase.

Similarly, with the known phase angle of mastic/mortar and asphalt binder, the interaction parameter of B can be determined by using the equation below:

(4) $$B=\left(\frac{{\tan\delta }_{m}}{{\tan\delta }_{c}}-1\right)/\left(1.5\emptyset_{f}\right),$$

According to Palierne model [15], the modulus of two-phase composite materials can be estimated by using Equation (5):

(5) $$G_{c}^{*}=G_{m}^{*}\times \left(\frac{1+1.5\emptyset_{f}C}{1-\emptyset_{f}C}\right),$$

where: $G_{C}^{*}$ and $G_{m}^{*}$ are the modulus of the composite material and the asphalt matrix, respectively; C is the interaction parameter that represents the matrix-particle reinforcement interaction. 

With respect to the system of asphalt mastic and mortar, the interaction parameter of C value can be determined by using the modulus of asphalt binder, mastic and mortar:

(6) $$C=\frac{\frac{G_{c}^{*}}{G_{m}^{*}}-1}{\left(1.5+\frac{G_{c}^{*}}{G_{m}^{*}}\right)\times \emptyset_{f}},$$

A greater C value indicates a stronger asphalt and mineral interaction. In Ziegel’s study [16], it was found the polymer-filler interaction parameter of C values determined from storage modulus ranged from 1.1 to 6.5 depending on the combination of polymer and filler, which indicated that C value is of fundamental importance in selecting fillers for various commercial end uses. 

An extensive research on asphalt-filler interaction ability done by Liu and Zhao, showed that the sensitivity of interaction parameters A, B, C from high to low is in the order of B, A and C. The change of B and A was complex while C tended to be relatively constant [14]. The C value determined by using complex modulus varied from 0.8 to 3.0 for asphalt mastics containing limestone, Portland cement and hydrated lime. Hydrated lime showed the strongest interaction between asphalt binder and filler, followed by Portland cement, and limestone had the least interaction among these three types of filler. The interaction ability of asphalt binder and filler increases with an increase in temperature and decreases with an increase in loading frequency. Guo’s study indicated that the interaction parameter C was the least sensitive compared with A and B yet effective to evaluate the effect of temperature, components of filler and specific surface area of filler on interfacial interaction. The C value ranged from 1.45 to 1.85 for mastic containing andesite, granite and limestone. Increasing temperature from 15 °C to 35 °C led to a higher C value. Guo concluded that temperature and the specific surface area of fillers were the two main factors effecting the interfacial interaction between asphalt binder and mineral fillers [15].

**Figure 9:**
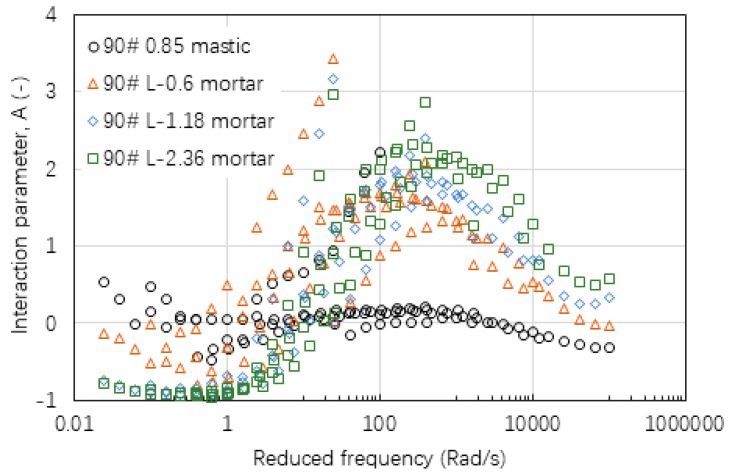
Interaction parameter of A values of mastic and mortar based on AC-25 mixture.

**Figure 10:**
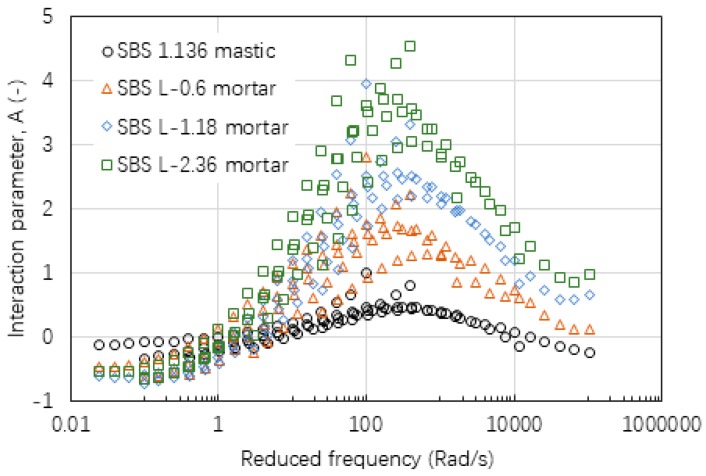
Interaction parameter of A values of mastic and mortars based on AC-20 mixture.

**Figure 11:**
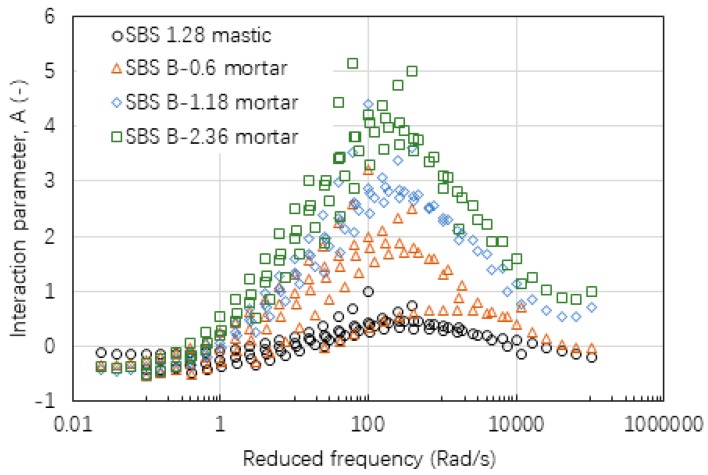
Interaction parameter of A values of mastic and mortar based on AC-13 mixture.

Figure 9, Figure 10 and Figure 11 gives the values of interaction parameter A for various types of mastic and mortar determined from various asphalt mixtures. According to these figures, A value changed significantly over a wide range of reduced frequency or temperature (30 °C to 70 °C). A peak value tended to occur at a reduced frequency of around 300 rad/min at a reference temperature of 60 °C. Asphalt mastic tended to have lower A value compared with the corresponding mortar, indicating that asphalt-filler interaction was stronger than those between asphalt binder and fine aggregate. Increasing the size of fine aggregates could result in higher A values, thus weak asphalt-aggregate interaction. It was found that neat asphalt-based materials in Figure 9 tended to have a lower A value than SBS modified asphalt-based materials in Figure 10. The difference between basalt and limestone fine aggregate is marginal as indicated in Figure 10 and Figure 11, respectively.

**Figure 12:**
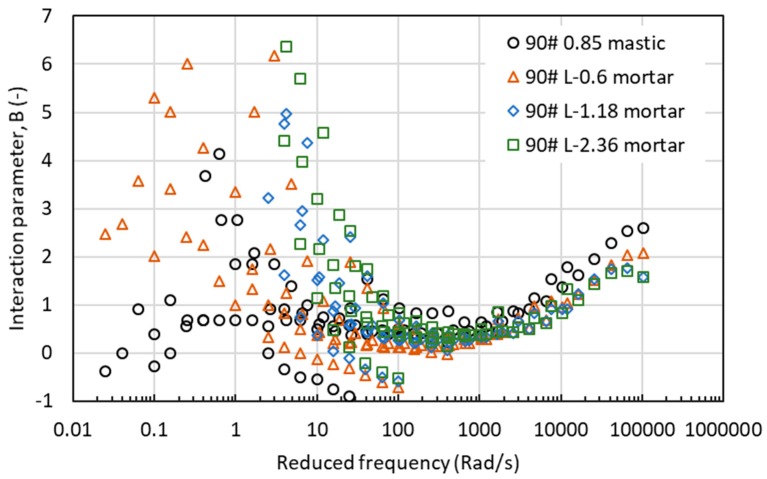
Interaction parameter of B values for mastic and mortar based on AC-25 mixture.

**Figure 13:**
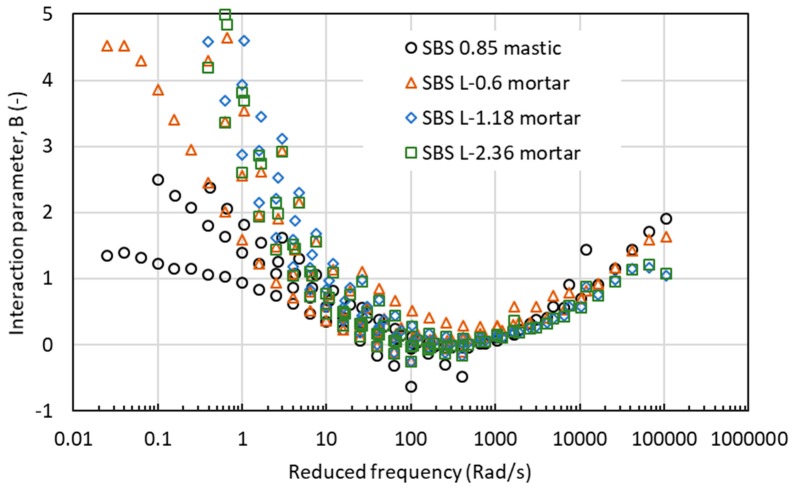
Interaction parameter of B values for asphalt mastic and mortar based on AC-20 mixture.

**Figure 14:**
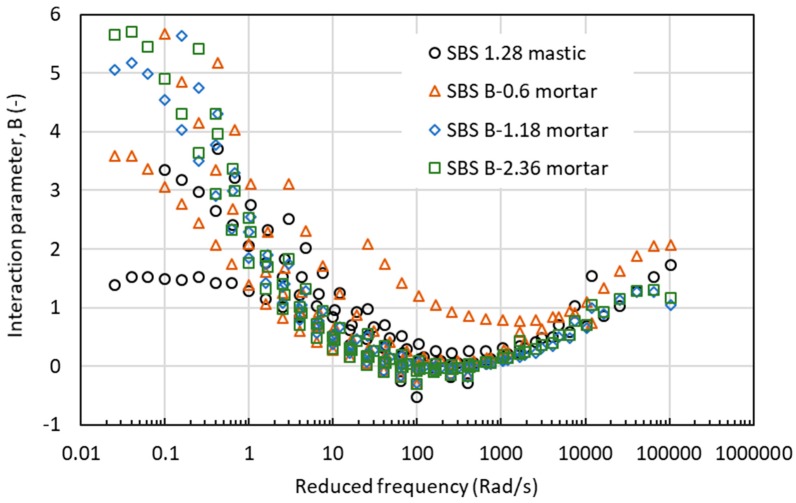
Interaction parameter of B values for asphalt mastic and mortar based on AC-13 mixture.

Figure 12, Figure 13 and Figure 14 show the values of interaction parameter B for various types of mastic and mortar. The change tendency of interaction parameter B was contrary to interaction parameter A. Increasing frequency or reducing temperature decreased the B value to a minimum at a frequency around 200rad/s with a reference temperature of 60 °C. After reaching this critical frequency, further increase in frequency resulted in an increase in B value. It was observed that B value could not distinguish well the difference between mastic and mortar as well the effect of aggregate size of mortar. Regardless of the type of asphalt material, larger scatter of B values was found at the low range of reduced frequency, or high temperatures. At high frequencies or low temperatures, asphalt mastic and mortar had a close value of interaction parameter B.

The analysis based on interaction parameters, A and B pointed to a phenomenon that asphalt-filler/aggregate interaction varies over a wide range of frequency or temperature (30 °C to 70 °C in this study). Furthermore, there was a critical frequency or temperature that may show poor interaction ability between asphalt binder and mineral particle. Since temperature has a great influence on asphalt binder’s viscoelastic properties, it indirectly hinted that asphalt-filler/aggregate interaction may be sensitive to the viscoelastic properties of asphalt binder.

**Figure 15:**
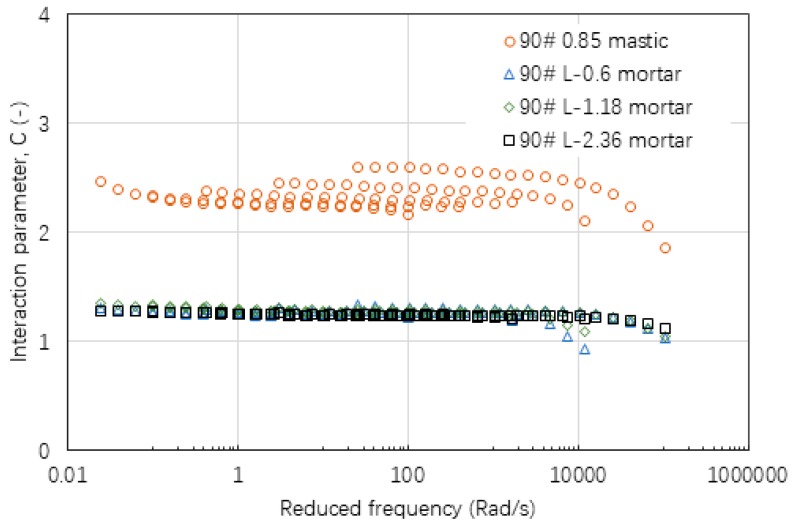
Interaction parameter of C values for asphalt mastic and mortar based on AC-25 mixture.

**Figure 16:**
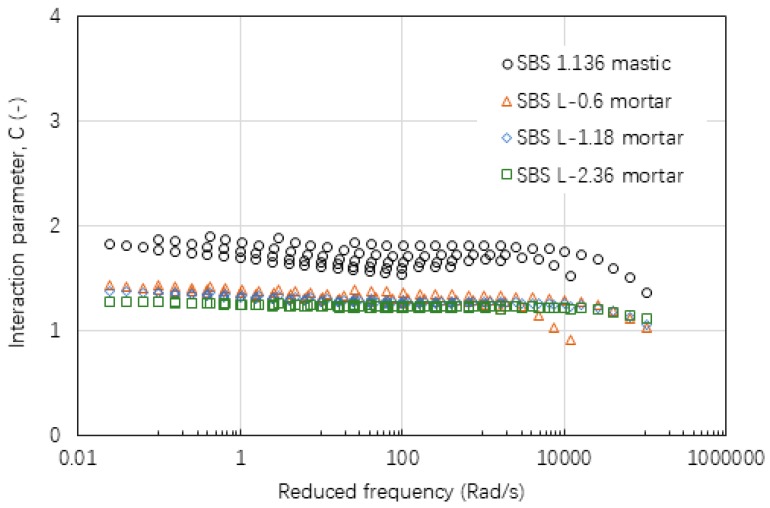
Interaction parameter of C values for asphalt mastic and mortar based on AC-20 mixture.

**Figure 17:**
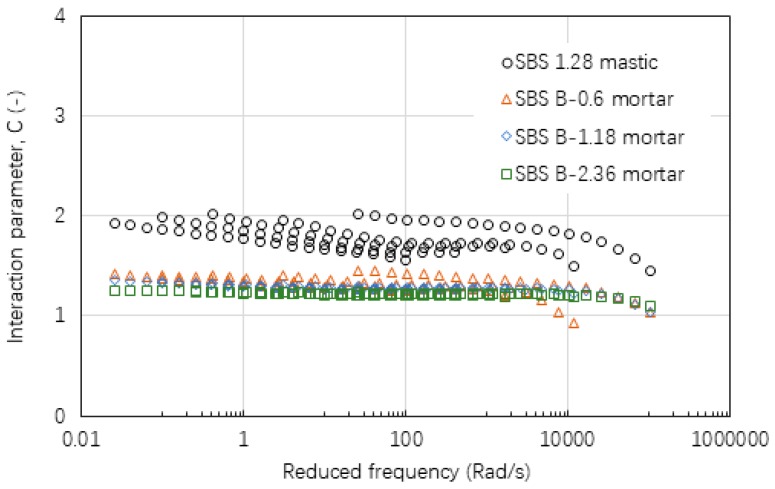
Interaction parameter of C values for asphalt mastic and mortar based on AC-13 mixture.

Figure 15, Figure 16 and Figure 17 show the values of interaction parameter C for various types of mastic and mortar. Compared with interaction parameters A and B, C tended to be relatively constant with a small change over a wide range of frequency or temperature. The higher value of C indicated that mastic had a stronger interaction ability compared with mortar. Bigger sizes of fine aggregate resulted in a very slight reduction on C value. Table 6 lists a summary of C values of various asphalt materials. Neat asphalt binder had a stronger interaction with filler compared with SBS modified asphalt binder. Increasing filler-asphalt binder ratio could slightly increase C value. All of the mortar tended to have a relatively constant C value ranging from 1.20 to 1.31 independent of the type of asphalt binder, aggregate and grading. 

**Figure 18:**
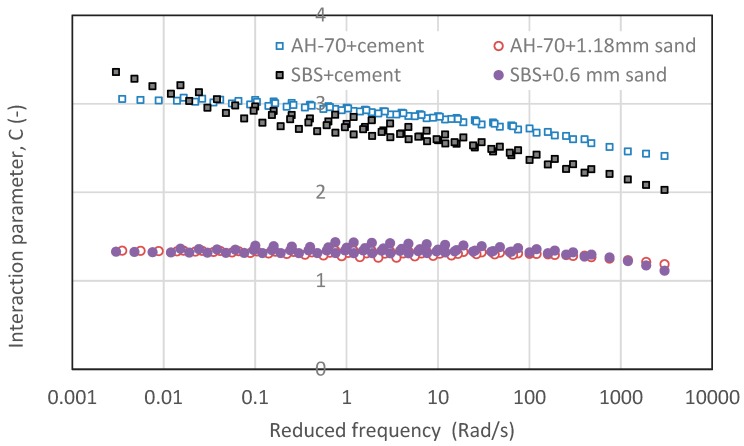
Interaction parameters, C values of mastic made by using cement as filler and mortar using river sand as a substitution for fine aggregate.

The above analysis indicated that the interaction parameters A and B were determined based on phase angle and they showed a large scatter due to frequency and temperature dependency. This may make the property prediction difficult. A relatively constant value of interaction parameter C was useful for modulus prediction. The validation of C value was carried out by using different materials and combinations. For this purpose, an AH-70 neat asphalt binder with a penetration of 67, Portland cement as filler as well as river sand as fine aggregate were applied. Two types of mastic were prepared by using AH-70 neat asphalt binder and SBS modified asphalt binder plus cement with a filler-asphalt binder ratio of 1:1. Two types of mortar were checked by AH-70 neat asphalt binder and SBS modified asphalt binder together with limestone filler and river sand as a substitution for fine aggregates. The compositions of these two types of asphalt mortar were determined according to the mortar with a maximum size of 1.18 mm and 0.6 mm based on AC-20 asphalt mixture as listed in Table 4. Figure 18 shows the tendency of C values of these four asphalt materials that were used for the purpose of validation. The C values of mastic containing cement tended to decline with increasing frequency while mortar containing river sand remained constant. Table 7 shows the validation results based on C values. It could be seen that AH-70 neat asphalt binder had a slightly higher C value compared with SBS modified asphalt binder. Similarly, cement filler had stronger interaction ability than limestone filler as listed in Table 6. Mortar containing river sand showed a comparable result with different types of mortar as listed in Table 6. It indicated that the interaction ability between asphalt binder and filler was stronger than those between asphalt binder and fine aggregate. The specific area of filler was found to significantly influence the interaction ability. In general, the C values remained relatively constant, independent of the type of materials and composition, thus could be better for the prediction of mortar modulus based on asphalt binder modulus. The above analysis indicated that the interaction between asphalt binder, filler and fine aggregate was a physical effect, which could be explained by particle filling effect. On the contrary with physical effect, the chemical interaction effect was reported by Singh in the crumb rubber-asphalt binder system [56]. Singh reported that the chemical interaction effect between crumb rubber and asphalt binder and the filler nature of rubber particles can be well identified. The effect of particle filler nature was found to be significantly greater than the effect of chemical interaction on viscosity, G*/Sinδ, recovery, and fatigue life, while the chemical interaction effect was obvious on nonrecoverable creep compliance response. Increased crumb rubber particle size may cause changes in the filler nature of crumb rubber modified asphalt composite. Compared with crumb rubber, it is clear that filler and fine aggregate lack chemical interaction with asphalt binder. Due to the simple particle filling effect, specific area could be significant for asphalt binder-filler mastic system, while the size of fine aggregate could be important for mortar system. Nevertheless, further testing could be carried out to reveal the microscopic bitumen-filler/aggregate interaction in the asphalt mixture composites. 

## 4. Conclusions

The rheological properties of asphalt binder, asphalt mastic and asphalt mortar were investigated by means of dynamic shear rheometer. The interaction between asphalt binder, mineral filler and fine aggregates was evaluated by using the data obtained from complex modulus and phase angle. Based on the experimental results and the analysis done, the following conclusions were drawn:

- The modulus reinforcement with the addition of filler and fine aggregates into asphalt binder can be well explained by particle reinforcement mechanics. Compared with asphalt binder, asphalt mastic could result in a three- to five-fold increase while asphalt mortar could have a modulus increase of one to two orders of magnitude. The phase change was identified by the significant reduction on phase angle at low frequencies or high temperatures. The combined effect of increasing modulus and reducing phase angle can contribute to high rutting resistance of asphalt mixtures.
- Three interaction parameters were used to analyze the interaction among asphalt binder, filler and fine aggregate. The sensitivity of these parameters was different and did not give a consistent result. The parameters based on phase angle showed large scatter; however, the parameter based on complex modulus was relatively constant.
- The interaction between the asphalt binder and the filler was stronger than its interaction with fine aggregates. Therefore, the specific area could be important for enhancing asphalt binder-filler interaction.
- Asphalt mortar tended to have a constant C value irrespective of type of asphalt binder, fine aggregate and material composition. This allowed for a better prediction of mortar modulus based on asphalt binder modulus. In general, the interaction between asphalt binder, filler and fine aggregate was physical and could be explained by particle filling effect. The temperature sensitivity of mastic and mortar was thus controlled by the type of asphalt binder. The effect of fine aggregate, for example, aggregate contact and friction on rheological properties was prominent at high temperatures. Considering the significant effect of modulus reinforcement and phase change, asphalt mortar could be a vital scale to bridge the gap between asphalt binder and asphalt mixture in a multiscale performance research.

## Figures and Tables

**Table 1:** Basic properties of the asphalt binders.

Index	Units	Asphalt Binder	
		Neat Bitumen (90#)	SBS Modified Bitumen
Penetration (25 °C, 100 g, 5 s)	0.1 mm	84	73
Ductility (15 °C, 5 cm/mm)	cm	98.1	>100
Viscosity (135 °C)	Pa·s	0.324	0.645
Softening point	°C	49.5	52

**Table 2:** Aggregate grading and compositions of various asphalt mixtures.

Sieve Size (mm)	Passing Percent (%)		
	AC-13	AC-20	AC-25
31.5	-	-	100
26.5	-	100	98
19	-	95	86
16	100	83	79
13.2	95	72	69
9.5	77	61	47
4.75	53	41	34
2.36	37	30	24
1.18	27	23	18
0.6	19	16	13
0.3	14	11	10
0.15	10	9	8
0.075	6	5	5
Aggregate	Basalt	Limestone	Limestone
Filler	Limestone	Limestone	Limestone
Asphalt binder	SBS	SBS	90#
Asphalt binder content	4.7	4.4	3.9

**Table 3:** Compositions of asphalt mastic contained filler and asphalt binder.

Specimen	Asphalt Binder (A)	Filler Type (F)	F/A Ratio
AC-13’s mastic	SBS	Limestone powder	1.280
AC-20’s mastic	SBS	Limestone powder	1.136
AC-25’s mastic	90#	Limestone powder	0.850

**Table 4:** Compositions of asphalt mortar containing find aggregates, filler and asphalt binder.

Sieve Size (mm)	Percent Composition by Weight (%)								
	Basalt Aggregates			Limestone Aggregates			Limestone Aggregates		
	I	II	III	I	II	III	I	II	III
2.36	27.7	-	-	24.2	-	-	22.4	-	-
1.18	18.2	25.2	-	16.5	21.8	-	16.2	20.8	-
0.6	13.0	18.0	24.0	14.3	18.9	24.2	16.2	20.8	26.3
0.3	9.5	13.2	17.6	11.0	14.5	18.6	12.4	16.0	20.2
0.15	6.1	8.4	11.2	5.5	7.3	9.3	6.2	8.0	10.1
0.075	6.9	9.6	12.8	7.7	10.2	13.0	8.7	11.2	14.2
Filler	10.4	14.4	19.2	11.0	14.5	18.6	8.3	10.6	13.4
Asphalt binder content	8.1	11.3	15.1	9.7	12.8	16.4	9.7	12.5	15.8
Asphalt binder	-	SBS	-	SBS			90#		
Asphalt mixture	AC-13			AC-20			AC-25		

Note: I, II and III are mortar scales containing fine aggregates with a maximum size of 2.36 mm, 1.18 mm and 0.6 mm respectively.

**Table 5:** Designed height of DSR test specimens.

Type of Binder	Height (mm)
Asphalt	1.0
Mastic	1.0
Mortar with fine aggregates smaller than 0.6 mm (0.6 mortar)	2.0
Mortar with fine aggregates smaller than 1.18 mm(1.18 mortar)	4.0
Mortar with fine aggregates smaller than 2.36 mm (2.36 mortar)	7.0

**Table 6:** A summary of interaction parameter, C values of various asphalt materials.

Material Scale	Asphalt Binder	Filler	Fine Aggregate	Interaction Parameter, C Value
Mastic	90#	Limestone (0.85:1)	-	2.523
	SBS	Limestone (1.136:1)	-	1.806
	SBS	Limestone (1.28:1)	-	1.877
Mortar	90#	Limestone	0.6 mm limestone	1.281
	90#	Limestone	1.18 mm limestone	1.272
	90#	Limestone	2.36 mm limestone	1.243
	SBS	Limestone	0.6 mm limestone	1.310
	SBS	Limestone	1.18 mm limestone	1.288
	SBS	Limestone	2.36 mm limestone	1.233
	SBS	Basalt	0.6 mm limestone	1.308
	SBS	Basalt	1.18 mm limestone	1.277
	SBS	Basalt	2.36 mm limestone	1.217

**Table 7:** Interaction validation by using cement as filler and river sand as a substitution for fine aggregate.

Material Scale	Bitumen	Filler	Fine Aggregate	Interaction Parameter, C Value
Mastic	AH-70	Cement (1:1)	-	2.864
	SBS	Cement (1:1)	-	2.678
Mortar	AH-70	Limestone	1.18 mm sand	1.312
	SBS	Limestone	0.6 mm sand	1.340
